# Competitive Formation Zones in Carbon Nanotube Float-Catalysis Synthesis: Growth in Length vs. Growth Suppression

**DOI:** 10.3390/ma15207377

**Published:** 2022-10-21

**Authors:** Vladimir Z. Mordkovich, Aida R. Karaeva, Nikita V. Kazennov, Eduard B. Mitberg, Mariem Nasraoui, Boris A. Kulnitskiy, Vladimir D. Blank

**Affiliations:** 1 Technological Institute for Superhard and Novel Carbon Materials, 7A Tsentralnaya street, Troitsk, 108840 Moscow, Russia; 2 Department of Electrochemistry, Faculty of Chemistry, M.V. Lomonosov Moscow State University, Leninskie Gory 1/3, 119991 Moscow, Russia

**Keywords:** carbon nanotubes, float-catalysis, nanocapsule, multishell carbon, TEM, maghemite

## Abstract

Catalytic synthesis of carbon nanotubes (CNT) produces numerous various byproducts such as soot, graphite platelets, catalyst nanoparticles, etc. Identification of the byproduct formation mechanisms would help develop routes to more selective synthesis of better carbon-based materials. This work reports on the identification of the formation zone and conditions for rather unusual closed multishell carbon nanocapsules in a reactor for float-catalysis synthesis of longer CNT. Structural investigation of the formed nanocapsule material along with computational fluid dynamics (CFD) simulations of the reactor suggested a nanocapsule formation mechanism, in which CNT embryos are suppressed in growth by the in-reactor turbulence. By means of TEM and FFT investigation, it is found that differently oriented single crystals of γ–Fe_2_O_3_, which do not have clear connections with each other, determine a spherical surface. The carbon atoms that seep through these joints do not form crystalline graphite layers. The resulting additional product in the form of graphene-coated (γ–Fe/Fe_3_C)/γ–Fe_2_O_3_ nanoparticles can be a lightweight and effective microwave absorber.

## 1. Introduction

Over the past two decades, researchers from different countries have done a significant amount of work to investigate the unique structural, electrical, mechanical, physicochemical, and other properties of carbon nanotubes (CNT). The valuable CNT properties provide a potentially wide scope for their application in biotechnology and biomedicine [1,2], electronics [3,4], composite materials [5,6,7], and other fields. Interest in CNT research has remained lively for at least 30 years and continues to this day. The mainstream research focuses on improving CNT quality and their application on an industrial scale.

A common problem in the CNT synthesis process is the formation of impurities such as amorphous carbon, catalyst nanoparticles, onion-like carbon, and other graphite nanostructures in addition to the main product. It is known [8,9] that the formation of additional impurities in the product depends on the method and conditions of synthesis. Among other methods reviewed in [8,9], the float-catalysis technique [10,11,12] is known as a basis for highly productive and continuous technology for CNT synthesis. Our state-of-the-art process of continuous longer CNT synthesis belongs to the float-catalysis family and produces, like other float-catalysis processes, some additional impurities and by-products [13]. It seems possible that at least some of these by-products are formed because of unsuccessful CNT embryonation, which can be located in specific zones of the float-catalysis reactor described in [13]. Deeper investigation of possible formation zones for either CNT elongation or CNT truncation is obviously in high demand.

The purpose of the present work is to investigate the additional carbon deposit (in particular bottom carbon deposit) and possible mechanisms of formation of these by-products and CNT.

In this regard, mathematical modeling of the flow trajectory of a feed gas in a CNT synthesis reactor and a study of the additional carbon deposit by means of transmission electronic microscopy (TEM) were carried out in this work. This analysis allows the suggesting of the mechanism of formation of rather unusual carbon nanocapsules in this deposit as an alternative route to CNT elongation.

## 2. Materials and Methods

### 2.1. Experimental Setup and Conditions

**Figure 1:**
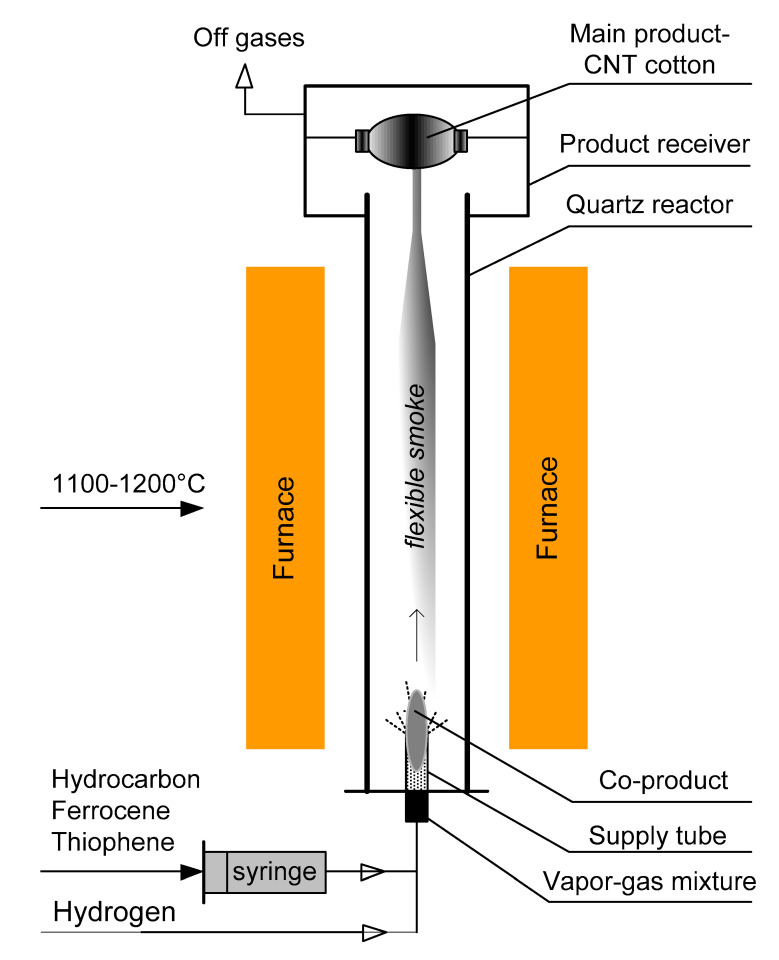
Schematic representation of the proprietary float-catalysis synthesis reactor rig.

The float-catalysis CNT synthesis method is based on decomposition of a carbon precursor and the CNT nucleation on the surface of floating active catalyst particles. Figure 1 represents schematically the CNT synthesis reactor rig. This figure depicts the main distinction of our synthesis technique, namely that the feed gas flow is directed from bottom to top, unlike other float-catalysis rigs described in the literature [10,11,12]. This is why the main synthesis product is collected as a spool of CNT in a product receiver at the top of the reactor. The bottom part of the reactor is highlighted in Figure 1 as a zone of preferable formation of an additional carbon deposit (bottom deposit). Ferrocene was used as a catalyst precursor, while thiophene was fed as a growth activator. The peculiarity of the CNT production method was that a reaction mixture of carbon precursors, thiophene activator, and a catalyst was fed into the reactor from bottom to top with a carrier gas flow through a feed injector tube (FIT) located in the bottom part of the reactor. In this work, carbon nanotubes were synthesized by the aerosol method, aka float-catalysis, at a temperature of 1100–1200 °C in a hydrogen gas carrier flow with ethanol/acetone as the carbon precursor. It is worth noticing that liquid components were fed into the flow of hydrogen carrier gas by means of a syringe pump. More details of the CNT synthesis method were published elsewhere [13].

Thorough measurement of temperature was done along the whole reactor by means of moving thermocouple probes. An important observation was done concerning the FIT zone, namely that although it is characterized by a relatively low temperature of 480–550 °C, that temperature is still higher than the 400–450 °C range, which is necessary for the decomposition of ferrocene and formation of floating Fe-catalyst nanoclusters.

Carbon nanotubes as a main product were observed to appear just atop of the FIT in the form of an aerogel or “flexible smoke”, which took immediately a shape of a hollow vertical stem pulled to the top of the reactor to get spooled into a cylindrical carbon-cotton bobbin.

The synthesis in the described setup results in the concentration of by-products in yet another deposit (additional carbon deposit or bottom carbon deposit) along the entire length of the FIT in the form of a thick layer. Sometimes, shapeless felt-like formations were found to get formed on the FIT top. This indicates that the feed gas mixture flow coming from the FIT creates a turbulence zone in the bottom part of the reactor, which is characterized by an intense reverse flow directed from the CNT growth zone with a temperature of more than 1000 °C back toward the FIT, where the temperature is less than 1000 °C. Thus, it provides an additional carbon deposit with completely different conditions of formation compared to the main product.

### 2.2. CFD Simulations Setup

Mathematical modeling of the flow trajectory of the gas mixture along the length of the reactor was performed using the SolidWorks Flow Simulation software module.

### 2.3. Samples Characterization

Identification of the deposits’ nanostructure including identification of unusual spherical nanocapsules was done using transmission electron microscopy (TEM). A JEM-2010 unit by JEOL (Tokyo, Japan) equipped with an energy-dispersive X-ray spectroscopy (EDS) attachment was used.

## 3. Results

### 3.1. CFD Simulations of the Synthesis Zone

**Figure 2:**
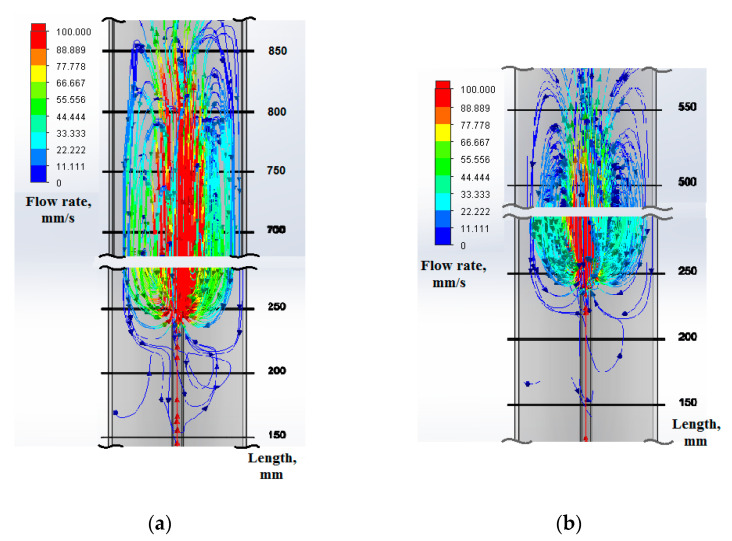
CFD simulation of the flow trajectory of a feed gas in the turbulence zone after exiting the FIT at two different feed gas flowrates: (**a**) hydrogen flowrate 2.8 L/min; (**b**) hydrogen flowrate 1.4 L/min.

CFD simulation of the feed gas flow pattern showed that an extensive turbulence appears at the exit of the feed gas from the FIT. Figure 2 shows the results of CFD simulation of the feed gas flow trajectory at different feed gas flowrates (without taking into account the thermal field). It can be seen from Figure 2a that a higher flowrate induces a larger turbulence area with much higher gas velocities inside. The turbulence area extends from the 200 mm mark up to the 900 mm mark, obviously involving the products occurring at the marks higher than 500 mm into a reverse flow with the probability to land them on the outer surface of FIT. Figure 2b manifests quite a different pattern, namely the turbulence area being much smaller and never extending beyond the 600 mm mark. Since the velocities are an order of magnitude lower, the probability of involving the products back to the outer surface of FIT should be lower.

Experimental observation of the FIT and the reactor inside after a synthesis run confirmed these suggestions. Indeed, the lower flowrate synthesis manifested uninterrupted growth of the main product stem, while a FIT post-synthesis review showed a moderate layer of additional deposit in the form of a thick loose coating, which is apparently a result of FIT-landing the intermediates intercepted from the appr. 500–600 mm mark area. The higher flowrate synthesis results in the formation of a much thicker additional deposit. On top of that, extensive flake-like formations grow at the edge of the FIT exhaust port. These flake-like formations interfere with the smooth CNT stem, making it irregular in shape and bringing to a complete disintegration after a 15–20 min run. The CFD simulation pattern suggests that at least part of reversed flow originates from the area up to mark 900 mm.

Due to CFD simulation, one can suppose that the additional carbon deposit originates from the turbulence zone or the stagnation zone. To answer this question, further experimental investigation is needed. Experimental observations show that the lower-flowrate synthesis produces a smaller amount of bottom deposit, thus confirming the correctness of the CFD simulation. Moreover, it was found experimentally that it is possible to avoid the formation of a bottom carbon deposit if the turbulence of the feed gas is suppressed or diminished by reducing the feed gas flowrate. Thus, this rather simple simulation helps in understanding the experimental results described beneath.

### 3.2. Identification of Multishell Carbon Nanocapsules and CNT Growth Mechanism Insight

The results of the bottom carbon deposit investigation showed the presence of large quantities of spherical particles of complex structure. In addition, some isolated short CNT were found, which look like a result of abrupt growth suppression.

**Figure 3:**
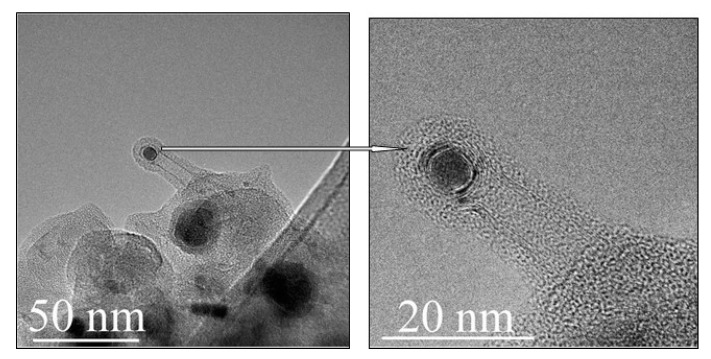
TEM images of a short single CNT as a co-product obtained in the turbulence zone of the CNT-cotton synthesis reactor.

Figure 3 shows a double-walled CNT, which managed to grow within a more intense reverse gas flow in the turbulence zone. As can be seen from Figure 3, CNT nucleation on the surface of the catalyst happened, but further growth was suppressed by encapsulation of an active catalyst nanoparticle (shown by an arrow in Figure 3), i.e., carbon atoms diffused in the catalyst volume in all directions, as a result of which the catalyst particle was covered with graphene layers and further CNT growth was suppressed.

It is important for understanding the process that reactive nanoparticles/clusters of iron are formed by ferrocene decomposition at a temperature of 500 °C and above as shown by the following equation:

Fe-(C_5_H_5_)_2_ → Fe + H_2_ + CH_4_ + C_x_H_y_ + … other reactive hydrocarbons

Those iron clusters acted as a catalyst, so both CNT and nanocapsules were presumably generated, growing because of active carbon atoms deposition until they are encapsulated.

An important role for increasing the rate of CNT nucleation on the surface of the catalyst is due to a sulfur-containing activator (thiophene). There is no sulfur-containing CNT growth activator in the low-temperature zone because the sulfur release temperature in the hydrogen stream is 800 °C for thiophene, while the turbulence zone is never heated beyond the temperature of 600 °C. As a result of the sulfur deficit in the lower temperature zones of the reactor, no CNT elongation occurs for the embryos deposited on the surface of FIT. Thus, we can observe discrimination of types of carbon nanostructures, i.e., the main product is dominated by longer CNT as reported in [13] in detail, while the additional (bottom) deposit is enriched with spherical nanocapsules and sort CNT, which is a result of growth suppression.

It is also necessary to note the role of the OH– radical for the CNT growth, which is formed in the high-temperature zone of the reactor because of ethanol decomposition. In the work [14], it is claimed that the OH– radical effectively removes amorphous carbon during the CNT growth, leaving only pure CNT as a product. The effect of etching by an OH– radical attacking carbon atoms is fully ongoing at a reaction temperature of 700–800 °C. Since the temperature in the turbulence zone is less than 600 °C, the effect of suppression of non-CNT carbon in the low-temperature zone is not observed.

Thus, the specific conditions for the formation of a bottom carbon deposit and the nucleation of nanostructures in the low-temperature zone of the reactor lead to the appearance of unusual carbon nanostructures. The elongation growth of these nanostructures, on the one hand, is interrupted because of the absence of a sulfur-containing activator and, on the other hand, because of the absence of an OH– radical, which would provoke the formation of other carbon impurities.

### 3.3. Characterization and TEM Analysis of Multishell Carbon Nanocapsules

**Figure 4:**
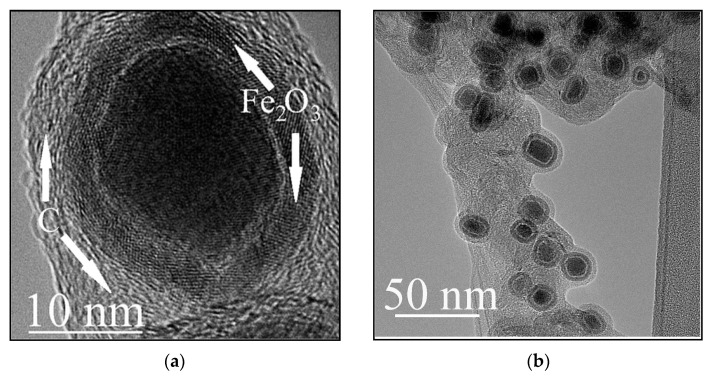

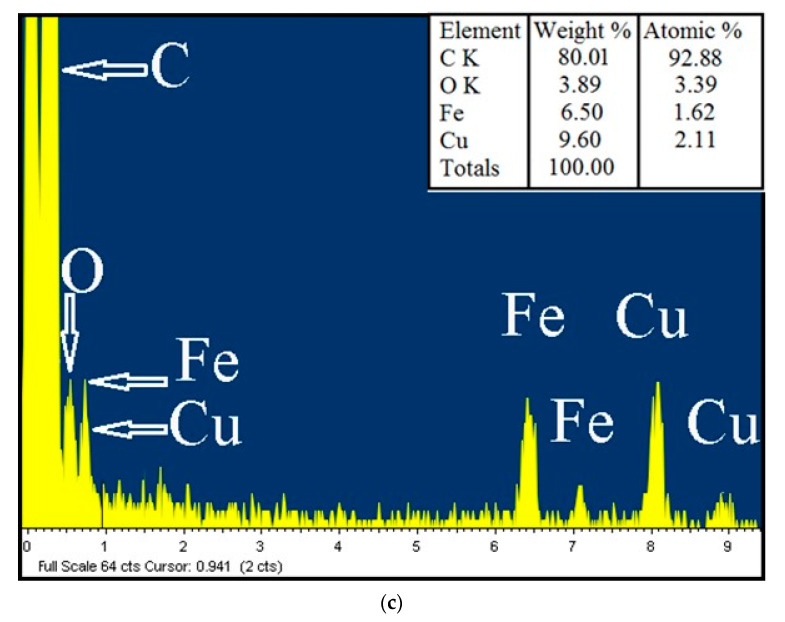
Fe- containing carbon nanocapsules: (**a**) general TEM view manifesting mean particle size 15–30 nm; (**b**) detailed structure of a nanocapsule by TEM (arrows indicate two encapsulating shells: inner Fe_2_O_3_ shell and outer carbon shell); (**c**) EDS spectra from view (**a**).

**Figure 5:**
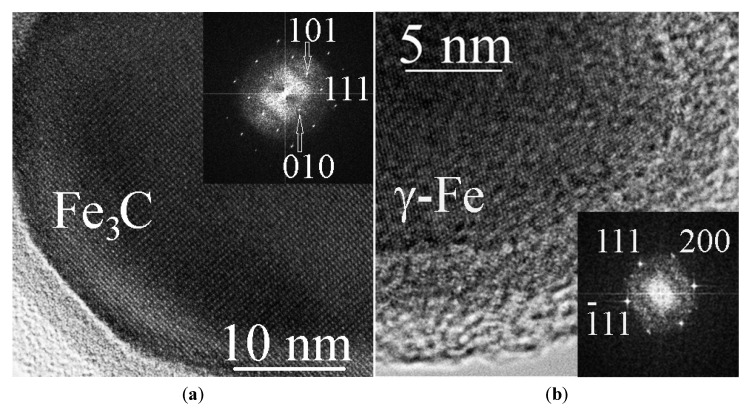
Fragments of nanocapsules, the inner part of which contains Fe_3_C carbide (**a**) and γ– Fe (**b**). The tabs show the corresponding FFT.

TEM studies have also shown the presence of complex-structure spherical particles in large amounts in the bottom carbon deposit sample. The size of such particles is on average 15–30 nm (Figure 4a), although there are particles of 50–80 nm in size that are also presented. Figure 4b shows a typical particle in which there are three layers: central, intermediate, and surface. Inside such a structure, there is a core containing iron. The middle layer contains iron and oxygen, while the outer layer contains only carbon. As a representative example, it can be seen from Figure 4b that, in total, the structure of the internal “nucleus” of 12 particles with a single-crystal structure was analyzed. In most cases, the structure corresponded to iron carbide-cementite (Fe_3_C)—10 times, 2 times the structure to γ– Fe. The corresponding structures, their fast Fourier transforms (FFT) images and transcripts are shown in Figure 5a,b.

The intermediate layer is a shell consisting of several single crystals that form a closed surface. Analysis showed that the intermediate shell contains layers of iron with oxygen with the structure of maghemite, γ– Fe_2_O_3_. Usually, the identification of maghemite causes difficulties in this analysis, since maghemite (γ– Fe_2_O_3_) and magnetite (Fe_3_O_4_) have cubic crystal lattices with similar parameters. Nevertheless, they can be distinguished. It is believed that the appearance of hk0 reflections arises from maghemite, which is seen to have cubic symmetry with a lattice parameter equal to that of magnetite but with a primitive Bravais lattice. This is associated with the order of vacancies [15,16].

**Figure 6:**
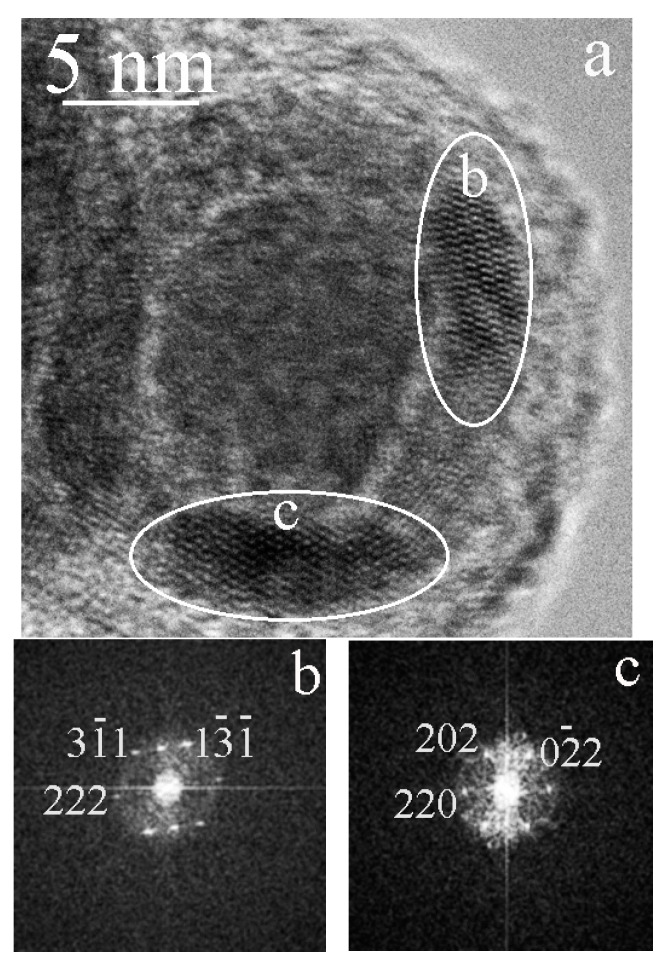
Carbon-encapsulated nanoparticles containing iron: (**a**) two fragments b and c are marked with white ellipses in the surface layer; (**b**,**c**) are the corresponding FFT, both fragments correspond to the cubic phase of γ– Fe_2_O_3_ (maghemite), the axes of zones [11-2] and [-111], respectively.

**Figure 7:**
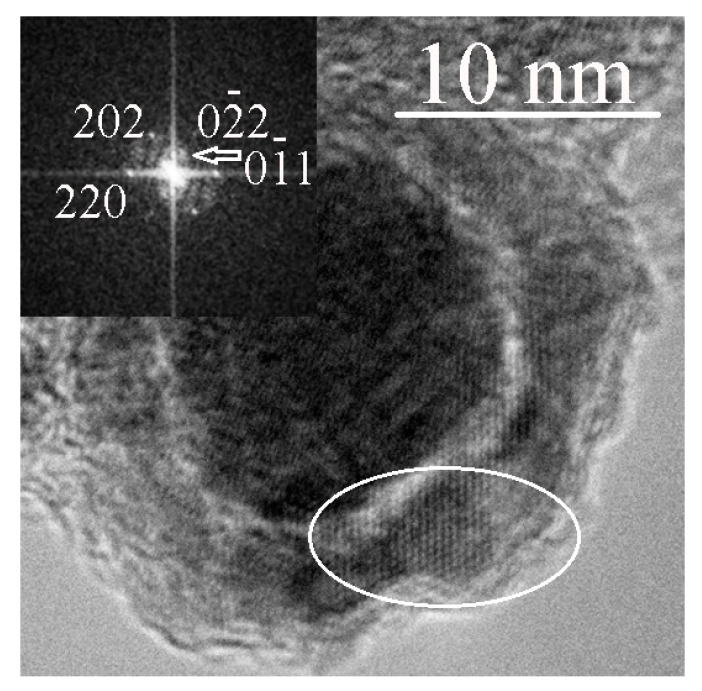
Carbon-encapsulated nanoparticles containing iron; a white ellipse marks a fragment with a γ– Fe_2_O_3_ lattice. The tab shows the FFT from the selected area. The presence of reflex (0–11) is characteristic of the maghemite lattice.

Figure 6a shows a particle in the surface layer of which two fragments b and c are marked with white ellipses. The corresponding FFT images are shown in Figure 6b,c. Both fragments correspond to the cubic phase of γ– Fe_2_O_3_ (maghemite) with the axes of zones [11-2] and [-111], respectively. Figure 7 shows a particle containing a fragment of a layer with the axis of the zone [1-1-1]. The presence of (0–11) reflex in Figure 7 indicates the structure of maghemite in the surface layer of particles.

**Figure 8:**
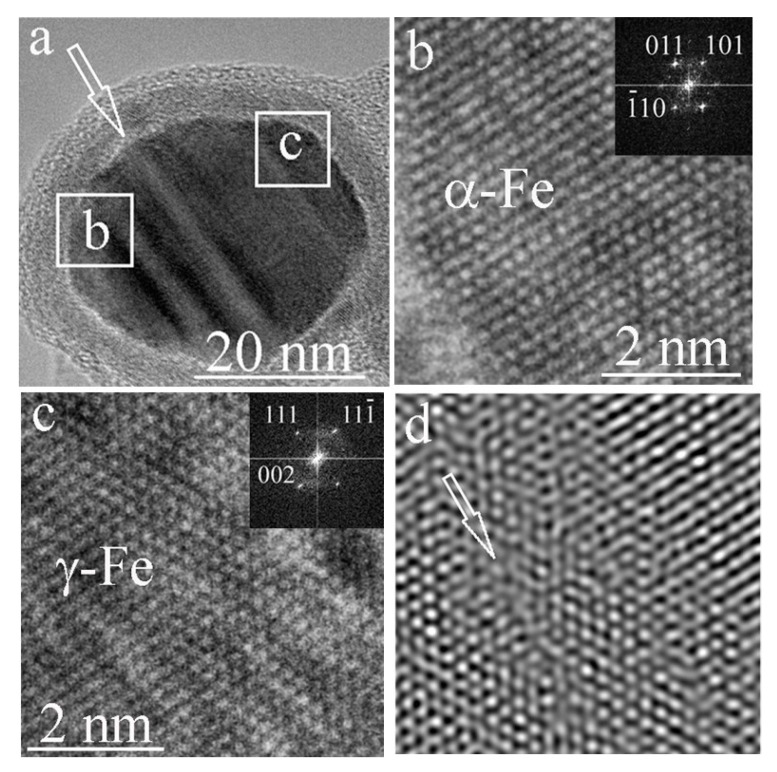
The particle through which the deformation bands pass (**a**). One of the bands is indicated by a white arrow; in the left part is the α– Fe (**b**), in the right part of the particle is the γ– Fe phase (**c**); (**d**) is the inverse FFT image of the deformation band (indicated by an arrow) with a structure close to amorphous.

Figure 8 shows a particle that differs from the previous ones. The particle has a complex structure. Deformation bands pass through the particle. One of these bands is indicated by a white arrow in Figure 8a. The left part of the inner particle is dominated by the α– Fe phase (Figure 8b), while the right part of the particle is dominated by the γ– Fe phase (Figure 8c). The stripes, judging by their structure, are deformational in nature. It can be seen from the FFT image in Figure 8d that the deformation bands have a structure close to amorphous.

The uppermost layer of the particle is curved graphite layers.

Particles of similar nature were obtained under different conditions. For example, under high pressure conditions, carbon-encapsulated iron carbide nanoparticles were synthesized in [17]. The material consists of the round carbide particles (5–40 nm) covered with onion-like carbon shells that are embedded into the carbon matrix. In another work, single-domain LaC_2_ microcrystals encapsulated within nanoscale polyhedral carbon particles have been synthesized in a carbon arc [18]. Typical particle sizes are on the order of 20 to 40 nm. Observation of the crystals indicates that LaC_2_ is protected from degradation by the carbon polyhedral shells of the nanoparticles. The encapsulation of many materials within a hollow graphitic cage was carried out using an electric arc discharge [19]. Iron particles (α– Fe, γ– Fe;) and a carbide phase were wrapped in graphitic carbon. The excellent protective nature of the outer graphitic cages against the oxidation of the inner materials was demonstrated. The synthesis method using an arc chamber to encapsulate controlled-size ferromagnetic transition metals, such as Fe, Co, and Ni, inside protective graphite shells was presented in [20]. There are works in which an oxygen-containing layer of iron was found. Iron nanoparticles embedded in an activated carbon matrix were synthesized in [21] by a pyrolysis process. The investigated structure exhibits an onion-like morphology that consists of a γ– Fe core surrounded by a double α– Fe (inner)/γ– Fe_2_O_3_ (outer) shell. Cubic and tetragonal modifications of maghemite were found within CNT, synthesized using the CVD method [22]. The outer shell, made of Fe_2_O_3_, was found on the surface of iron carbide particles in carbon nanofibers obtained by the CVD method in the presence of an Fe catalyst [23]. There are also thin heterogeneous Fe_2_O_3_/Fe_3_C-graphene layers for charge-storage amplification in Li-ion batteries obtained via the CVD method in two steps. The first step was to fabricate the nanoporous C-Fe_2_O_3_ layer; then a few graphene layers were grown on Fe_2_O_3_/Fe_3_C [24].

The features of our method for forming three-layer particles are as follows. Both carbon and oxygen are taking part in the Fe/Fe_3_C particles growing and forming process. Both diffuse through the particle up to its surface. Oxygen, which reaches the surface, combines with iron and forms a shell consisting of iron oxide. Further diffusion of carbon through the shell is difficult and since there is no back diffusion to Fe_3_C possible, there is a possible reaction with iron oxide with CO and CO_2_ formation. Those gases may also serve as a barrier for iron ions to diffuse outward to the oxide layer [25]. It is known that when iron interacts with oxygen, according to the iron–oxygen phase diagram, the formation of different oxides is possible. The iron oxide film may contain iron monoxide (FeO), which corresponds to the lowest oxygen content in the oxide, followed by an intermediate layer of Fe_3_O_4_ and on the outer surface of the film, γ– Fe_2_O_3_ [26]. In the present work, the first two phases were not detected. The shape of the iron particle is close to spherical, whereas its structure is crystalline. Therefore, oxygen atoms reach the surface of the particle on different sides of the crystal, leading to the formation of thin, differently oriented crystal fragments of γ– Fe_2_O_3_ on the surface. Thus, a spherical surface is formed of differently oriented single crystals of γ– Fe_2_O_3_. These fragments do not have clear connections with each other. Therefore, there is no real crystal lattice at the crystal junctions, which can contribute to further carbon diffusion. The carbon atoms that seep through these joints do not form crystalline graphite layers. The surface turns out to be covered with distorted graphite fragments that are close to amorphous.

The deformation bands shown in Figure 8 indicate the processes that accompanied the formation of the inner particle. Such shear bands (regions in which inelastic shear deformation is localized and exceeds the deformation in surrounding material) are found in many materials: rocks, ceramics [27], polymers, fullerenes [28], polycrystalline alloys, metal glasses, and metals [29]. In our case, it can be argued that at least some particles were subjected to severe deformation during the formation process.

Studies by the authors [30,31] have shown that graphene-coated Fe nanoparticles have a good ability to absorb electromagnetic waves. Because of the charge transfer at the Fe–graphene interface, graphene-coated Fe nanoparticles exhibit excellent dielectric properties that result in an excellent microwave absorption capacity over a wide frequency range. Thus, the resulting additional product in the form of graphene-coated (γ– Fe/Fe_3_C)/γ– Fe_2_O_3_ nanoparticles can be a lightweight and effective microwave absorber.

## 4. Conclusions

The formation zone and conditions for rather unusual, closed multishell carbon nanocapsules were identified in a reactor for float-catalysis synthesis of longer CNT. Structural investigation of the formed nanocapsule material along with CFD simulations of the reactor suggested a nanocapsule formation mechanism, in which CNT embryos are suppressed in growth by the in-reactor turbulence. In the course of TEM studies of CNT synthesis products obtained by the CVD-method, closed multilayer carbon nanocapsules with unusual structure were discovered. This product was formed in the low-temperature zone of the reactor in the form of an additional deposit, as was confirmed by the mathematical modeling simulation. The study of its structure gave an idea of the mechanism of CNT formation, interrupted by the trajectory of the steam-gas flow in the turbulence zone of the reactor. The multi-shell structure of spherical shape is framed with disordered carbon, the intermediate layer of γ– Fe_2_O_3_ is also kept spherical, and, as confirmed, the carbonization of the catalyst particle occurs by diameter, since Fe_3_C or γ– Fe are contained inside the nanocapsule. The results of this research pave the way for prospective experimental methods allowing cleaner CNT formation. In addition, the resulting additional product in the form of graphene-coated (γ– Fe/Fe_3_C)/γ– Fe_2_O_3_ nanoparticles can be a lightweight and effective microwave absorber.

## Data Availability

Not applicable.
